# Perfil de Morte por Todas as Causas e o Comportamento das Doenças do Aparelho Circulatório na Infância entre 2019 e 2022 no Brasil

**DOI:** 10.36660/abc.20250191

**Published:** 2025-12-22

**Authors:** Thayanne Mendes de Andrade, Mariara Lopes da Costa Marques, Julia Pereira Cavalcante Marques, Maria Eduarda Miranda de Souza, Gláucia Maria Moraes de Oliveira, Thais Rocha Salim

**Affiliations:** 1 Universidade Federal do Rio de Janeiro Rio de Janeiro RJ Brasil Universidade Federal do Rio de Janeiro, Rio de Janeiro, RJ – Brasil; 2 Universidade de Vassouras Vassouras RJ Brasil Universidade de Vassouras, Vassouras, RJ – Brasil

**Keywords:** Doenças Cardiovasculares, Mortalidade da Criança, Parada Cardíaca

## Abstract

**Fundamento:**

Entre 2020 e 2023, a pandemia da COVID-19 impactou diretamente a saúde da população. No Brasil, faltam estudos sobre correlação entre mortalidade por doenças do aparelho circulatório (DAC) e parada cardiorrespiratória (PCR) em menores de 20 anos nesse período.

**Objetivo:**

Analisar as taxas de mortalidade (TxM) por causas básicas de morte em menores de 20 anos no Brasil de 2019 a 2022, considerando DAC e PCR, para entender o impacto da pandemia.

**Métodos:**

Estudo ecológico de série temporal entre 2019 e 2022, focando nos óbitos de menores de 20 anos. Analisaram-se as TxM e a mortalidade proporcional (MP) por causa de óbito, com dados obtidos do DATASUS. Utilizou-se STATA e Excel para análise estatística.

**Resultados:**

Entre 2019 e 2022, ocorreram 243.358 óbitos em menores de 20 anos, com uma TxM de 101,7 por 100 mil habitantes, sendo maior entre os menores de 1 ano. As principais causas de morte foram perinatais, externas e malformações congênitas. Observou-se redução de 9% na TxM entre 2019 e 2020, seguida de aumento de 4,08% entre 2021 e 2022, e queda de 10% no número de nascidos vivos. As maiores TxM foram nas regiões Norte e Nordeste. Embora a TxM por DAC tenha diminuído entre 2019 e 2021, houve aumento em 2022. A descrição de PCR como causa de óbito foi pouco expressiva.

**Conclusão:**

Houve redução na TxM por todas as causas e aumento por DAC em maiores de 15 anos durante a pandemia, destacando a disparidade nas regiões de menor renda.

## Graphical abstract

**Figura Central:**
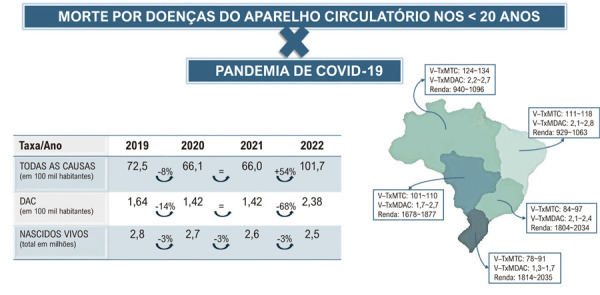
Perfil de Morte por Todas as Causas e o Comportamento das Doenças do Aparelho Circulatório na Infância entre 2019 e 2022 no Brasil

DAC: doença do aparelho circulatório; V-TxMTC: variação da taxa de mortalidade por todas as causas entre 2019 e 2022; V-TxMDAC: variação da taxa de mortalidade por DAC entre 2019 e 2022; Renda: renda per capita em reais.

## Introdução

Nas últimas duas décadas, as taxas de mortalidade (TM) diminuíram em todo o mundo entre pessoas com menos de 20 anos. O Brasil apresentou tendência semelhante, com redução de mais de 50% nas TM. Essa trajetória pode ser atribuída à melhoria do acesso à saúde e à educação, bem como à redução das taxas de pobreza e de fecundidade.^1^No entanto, as causas externas de morte, ou seja, aquelas causadas não por doenças, mas por fatores ambientais e perinatais, ainda são as principais causas básicas de morte no Brasil. Essas causas poderiam ser prevenidas ou atenuadas por meio de melhorias na assistência a gestantes e recém-nascidos, juntamente com medidas de saúde pública e educação para diminuir a desigualdade social e reduzir essas mortes.

Entre 2020 e 2023, a pandemia da COVID-19 afetou diretamente a saúde da população, mudando não só a forma como vivemos, mas também como adoecemos e morremos.

Um estudo ecológico, multicêntrico, incluindo 18 países de baixa e média renda demonstrou que, no período de 2020 a 2021, ocorreu um aumento de 3,6% na mortalidade infantil e de 1,5% na mortalidade materna, o que os autores correlacionaram à diminuição de acesso à saúde e ao diagnóstico precoce para possível tratamento.^2^ Em contrapartida, um estudo de coorte realizado na Inglaterra, país considerado de alta renda, no mesmo período, demonstrou uma redução na mortalidade infantil, o que foi correlacionado à queda das taxas de morte por doenças infecciosas, provavelmente causada pelo distanciamento social e medidas de prevenção da COVID-19 as quais reduzem também as doenças infecciosas respiratórias.^3^ Dessa forma, é possível perceber uma diferença de comportamento das taxas de mortalidade infantil de acordo com o perfil socioeconômico dos países e, possivelmente, com os seus modos de enfrentamento à pandemia.

No Brasil, não encontramos estudos de correlação entre taxa de mortalidade infantil e comportamento das causas básicas de morte nos menores de 20 anos, no período da pandemia de COVID-19, e níveis socioeconômicos dos seus municípios. Nossa hipótese é que o isolamento e as restrições sociais, embora necessários para conter a disseminação do coronavírus, podem ter levado à redução do acesso à saúde no país, com efeito prejudicial ao diagnóstico e tratamento de doenças, principalmente aquelas que exigem mais recursos e pessoal para diagnóstico, como as doenças do aparelho circulatório (DAC), resultando no aumento de óbitos nessa faixa etária no período.

Ainda, a doença cardiovascular foi observada como um dos principais fatores de risco para pior prognóstico e para evolução a óbito na COVID-19.^4-10^ No Brasil, em adultos, se observou que as doenças do aparelho circulatório como causa básica de óbito e a descrição da parada cardiorrespiratória (PCR) como causa de morte diminuíram no período da pandemia, fato que possivelmente esteve relacionado à redução no acesso ao diagnóstico, tratamento e possivelmente ao maior relato da COVID-19 como causa básica de óbito.^11^ Não foram encontrados estudos no país que tenham avaliado o comportamento das mortes por doenças do aparelho circulatório em crianças e adolescentes durante a pandemia.

Sendo assim, o objetivo desse estudo é realizar uma análise populacional das taxas de mortalidade por causas básicas de morte no Brasil nos menores de 20 anos, nos anos de 2019, 2020, 2021 e 2022, em ambos os sexos, antes e durante a pandemia de COVID-19, e seu comportamento nas regiões federativas do país. Além disso, avaliar o perfil de morte por doenças do aparelho circulatório (DAC) no mesmo período e faixa etária, e a descrição de PCR como causa básica de óbito nas declarações de óbito (DO), a fim de entender o impacto da pandemia na forma de morrer na infância.

## Métodos

### Desenho do estudo

Estudo ecológico de série temporal, de 2019 a 2022, de óbitos em menores de 20 anos, no qual se avaliaram as taxas de mortalidade e mortalidade proporcional por causa básica de óbito, em ambos os sexos no Brasil. Foram analisadas a distribuição por quartis das taxas de morte por causas básicas, a taxa de mortalidade por DAC e a descrição da PCR nas DO, na mesma faixa etária e período, nas regiões federativas do país, além da tendência de nascidos vivos, renda per capita, número de estabelecimentos de saúde por habitante e a taxa de mortalidade por todas as causas. O estudo foi realizado de acordo com princípios éticos, e por se tratar de bancos de dados nacionais não identificados, disponíveis no sítio do DATASUS, foi dispensada a aprovação pelo comitê de ética e pesquisa, seguindo a resolução 466/2012.

### Origem dos dados

Os dados sobre os óbitos foram obtidos no sítio do Departamento de Informática do Sistema Único de Saúde (DATASUS) por meio do Sistema de Informação sobre Mortalidade (SIM).^4^ Estas informações são compostas pelos conjuntos de todas as DO registradas no Brasil, de 2019 a 2022, ano a ano, por cada estado da federação. Foi utilizada a codificação de causa básica de óbito de acordo com a 10^a^ Revisão da Classificação Estatística Internacional de Doenças e Problemas Relacionados à Saúde, da Organização Mundial da Saúde (CID 10).^8^ Todos os arquivos foram convertidos para análise a partir do software Tab para Windows versão 4.15, do DATASUS. Esses dados foram coletados em ambos os sexos e nas seguintes faixas etárias: (1) menores de 1 ano (2) de 1 a 4 anos, (3) de 5 a 9 anos, (4) de 10 a 14 anos e (5) de 15 a 19 anos, seguindo o padrão proposto pela Organização Mundial da Saúde.^5^

As informações referentes às populações, utilizadas para produzir as taxas de mortalidade, são projeções derivadas de cálculos estatísticos realizados pelo Instituto Brasileiro de Geografia e Estatística (IBGE),^6^ com base nos censos, e estão disponíveis de 1980 a 2050 por macrorregião brasileira, sexo, faixa etária e pelos totais. Foram utilizadas no estudo as projeções de 2019 a 2022, nas faixas etárias 0-4 anos, excluídos os nascidos vivos do período, 5-9 anos, 10-14 anos e 15 a 19 anos, em ambos os sexos e em cada estado do Brasil. Para os menores de um ano foi utilizado o número de nascidos vivos disponíveis no Sistema de Informações sobre Nascidos Vivos (SINASC).^7^

As informações sobre renda per capita foram obtidas no sítio do IBGE, a partir da pesquisa nacional por amostra de domicílios contínua, realizada em 2022, ano a ano, entre 2019 e 2022, e por região federativa.

Os dados sobre estabelecimentos de saúde foram do sítio do DATASUS, por meio do Cadastro Nacional de Estabelecimentos de Saúde (CNES), exibidos no TABNET, ano a ano, entre os anos de 2019 e 2022, por região federativa. Esses estabelecimentos prestam atendimento a todas as faixas etárias sem distinção.

### Análise dos dados

Foram calculadas as taxas de mortalidade (TxM) por 100 mil habitantes e a mortalidade proporcional (MP) por causa básica de óbito nos menores de 20 anos, por sexo e faixa etária, nos anos de 2019, 2020, 2021 e 2022. Também foram calculadas as taxas de mortalidade por causa básica de óbito nos menores de 20 anos, por sexo e faixa etária, distribuídos nas regiões federativas Norte, Nordeste, Sul, Sudeste e Centro-oeste, e seus quartis, apresentados em tabelas. A PCR teve sua descrição como causa básica de óbito calculada, bem como sua TxM e MP, e apresentada em tabela a fim de análise de sua ocorrência, apesar de ser considerada como *garbage code.*

Os quartis foram calculados ano a ano a partir do percentil das taxas de mortalidade nos menores de 20 anos, por causas básicas de morte, no Brasil e em cada região federativa. São divididos entre as 25%, 50%, 75% e 100% menores taxas de mortalidade, no período, e devem ser analisados ano a ano.

Também foram analisadas separadamente as taxas de mortalidade por doenças do aparelho circulatório no Brasil e suas regiões, em ambos os sexos, distribuídas por faixas etárias, bem como a tendência de nascidos vivos nesse mesmo período e a taxa de mortalidade por todas as causas básicas de morte nos menores de 20 anos.

Foi construído um modelo de análise estatística em PanelOLS com dados fixos, utilizado para regressão de séries temporais. A variável dependente foi a taxa de mortalidade por todas as causas em menores de 20 anos por macrorregião brasileira ano. Enquanto a variável independente foi a renda per capita por macrorregião e ano. Com essas informações, podemos inferir que o objetivo da análise é investigar o efeito da renda per capita sobre a taxa de mortalidade em menores de 20 anos nas diferentes regiões do Brasil, levando em consideração as características fixas de cada região e a evolução temporal dos dados. A variável independente foi a renda per capita.

Os programas operacionais utilizados foram o Excel-Microsoft^8^ e Stata^®^ versão 14.^9^

## Resultados

Como é mostrado na Figura Central, no Brasil de 2019 a 2022, ocorreram 243.358 óbitos em menores de 20 anos por todas as causas básicas de morte, em ambos os sexos, com taxa de mortalidade de 101,7 por 100 mil habitantes no período e com maiores taxas de óbito entre os menores de 1 ano de vida, independentemente da causa básica de morte. As principais causas de morte foram perinatais, externas e malformações congênitas, respectivamente. A taxa de mortalidade apresentou uma queda de 9% entre os anos de 2019 e 2020 e de 0,1% entre os anos de 2020 e 2021, com um aumento de 4,08% entre 2021 e 2022, ocorrendo uma redução de 10% no número de nascidos vivos durante todo o período. As maiores taxas de mortalidade, ocorreram nas regiões Norte e Nordeste. A taxa de mortalidade por doenças do aparelho circulatório, no geral, sofreu redução entre 2019 e 2021 no Brasil e suas regiões com algumas exceções, com posterior aumento no ano de 2022. A descrição de PCR na DO durante todo o período não se mostrou expressiva em todas as faixas etárias, com TxM variando entre 0,01 e 0,03 por 100 mil habitantes no período.

A taxa de mortalidade e a mortalidade proporcional no Brasil por faixa etária e causa básica de morte pode ser visualizada na Tabela 1. Nos quatro anos de observação, as principais causas de morte foram perinatais, externas e malformações congênitas, com a maior TxM sendo de 1238,9 por 100 mil habitantes no ano de 2019 por todas as causas nos menores de 1 ano, com progressiva queda nos anos seguintes, sendo as malformações congênitas a segunda condição com maior taxa de mortalidade nesta faixa etária. A segunda faixa etária com maior taxa de mortalidade é a de 15 a 19 anos, se destacando a TxM de 105,3 por 100 mil habitantes no ano de 2020 por todas as causas básicas de óbito, com aumento quando comparada ao ano de 2019, tendo as causas externas como maior relevância. Em todas as outras faixas etárias a principal causa de morte foi a externa, com exceção do ano de 2022, que apresentou as doenças respiratórias como principal causa de morte na faixa etária de 1 a 4 anos.

**Tabela 1:** – Causas básicas de óbito, por faixas etárias, em menores de 20 anos, nos períodos de 2019 a 2022, em ambos os sexos no Brasil

Causas básicas de óbito		2019						2020						2021						2022					
		TOTAL	< 1	1-4	5-9	10-14	15-19	TOTAL	<1	1-4	5-9	10-14	15-19	TOTAL	<1	1-4	5-9	10-14	15-19	TOTAL	<1	1-4	5-9	10-14	15-19
**Infecciosas**	Óbitos MP TxM	2.512 4 3	1.263 4 44	467 8 4	202 6 1	198 5 1	382 2 2	3.086 5 3	1.381 4 50	537 12 4	247 9 1	271 7 2	650 4 4	3.484 6 4	1.432 4 53	570 11 5	254 9 2	341 9 2	887 6 6	3.406 5 6	1.608 5 55	688 11 6	288 9 2	305 7 2	517 3 3
**Neoplasias e sangue**	Óbitos MP TxM	3.312 5 4	326 1 11	663 11 6	683 22 4	670 16 4	970 6 6	2.889 5 3	251 1 9	553 12 4	570 22 4	645 16 4	870 5 5	3.029 5 3	286 1 11	625 12 5	612 22 4	610 16 4	896 6 6	3.041 5 5	291 1 10	654 10 5	613 19 4	616 15 4	867 6 5
**Endócrinas e Sistema nervoso**	Óbitos MP TxM	3.560 5 4	753 2 26	783 13 7	550 17 4	610 14 4	864 5 5	2.911 5 3	646 2 23	542 12 4	347 13 2	555 14 4	821 5 5	3.041 5 3	639 2 24	653 13 5	411 15 3	514 13 3	848 5 5	3.755 6 6	693 2 24	857 13 7	563 17 4	639 16 4	1.003 7 6
**Sistema respiratório**	Óbitos MP TxM	3.694 6 4	1.627 4 57	1.013 17 9	277 9 2	300 7 2	477 3 3	2.165 3 2	829 2 30	479 10 4	164 6 1	203 5 1	490 3 3	2.302 4 3	1.012 3 38	524 10 4	179 6 1	188 5 1	399 2 2	3.951 6 6	1.600 5 55	1.232 19 10	317 10 2	298 7 2	504 3 3
**Perinatal**	Óbitos MP TxM	20.291 31 23	20.213 57 709	42 1 0	14 0 0	7 0 0	15 0 0	18.665 31 21	18.618 59 682	24 0 0	8 0 0	3 0 0	12 0 0	18.510 31 21	18.468 58 691	24 0 0	8 0 0	2 0 0	8 0 0	17.935 30 30	17.869 55 617	31 0 0	11 0 0	9 0 0	15 0 0
**DAC**	Óbitos MP TxM	1.476 2 2	344 1 12	219 4 2	137 4 1	236 6 1	540 3 3	1.267 2 1	279 1 10	154 3 1	104 4 1	181 4 1	549 3 3	1.261 2 1	270 1 10	164 3 1	121 4 1	211 5 1	495 3 3	1.413 2 2	289 1 10	207 3 2	160 5 1	241 6 1	516 3 3
**MC**	Óbitos MP TxM	9.799 15 11	8.396 24 295	812 14 7	212 6 1	186 4 1	193 1 1	8.318 14 9	7.273 23 266	594 13 5	149 6 1	153 4 1	149 1 1	8.374 14 9	7.311 23 273	621 12 5	177 6 1	117 3 1	148 1 1	8.816 14 15	7.399 23 255	772 12 6	254 8 2	180 4 1	211 1 1
**Externas**	Óbitos MP TxM	16.310 25 18	981 3 34	1.209 21 11	736 23 5	1.549 36 9	11.835 71 70	16.023 27 18	863 3 31	1213 26 10	752 29 5	1.445 37 10	11.750 70 74	14.797 25 16	1.034 3 39	1.249 25 10	708 25 5	1.353 36 9	10.453 67 67	14.107 23 24	1.183 3 41	1.208 19 10	711 22 5	1.313 32 9	9.692 67 63
**Outras causas**	Óbitos MP TxM	1.847 3 2	500 1 17	258 4 2	184 6 1	244 6 4	661 4 4	1.559 2 2	469 1 17	215 4 2	122 5	208 5 1	545 3 3	1.675 3 2	404 1 15	253 5 2	135 5 1	233 6 1	650 4 4	1.804 3 3	486 1 17	294 4 2	176 5 1	261 6 2	587 4 4
**Mal definidas**	Óbitos MP TxM	2.405 4 3	890 2 31	356 6 3	171 5 1	224 5 1	764 4 4	2.246 4 2	830 2 30	275 6 2	119 4 1	216 5 1	806 5 5	2.262 4 2	874 3 33	302 6 2	163 6 1	208 5 1	715 4 4	2.070 3 3	839 2 29	340 5 3	157 5 1	190 4 1	544 4 3
**PCR**	Óbitos MP TxM	7 0 0	0 0 0	2 0 0	0 0 0	1 0 0	4 0 0	20 0 0	7 0 0	1 0 0	0 0 0	3 0 0	9 0 0	20 0 0	7 0 0	0 0 0	2 0 0	3 0 0	8 0 0	28 0 0	5 0 0	4 0 0	3 0 0	3 0 0	13 0 0
**TOTAL**	Óbitos MP TxM	65.213 100 107	35.293 100 1226	5.824 100 47	3166 100 20	4225 100 27	16.705 100 100	59.149 100 98	31.446 100 1117	4.587 100 37	2.582 100 17	6.465 100 24	16.651 100 98	58.755 100 98	31.737 100 1076	4.985 100 42	2.770 100 19	3.780 100 25	15.507 100 99	60.326 100 102	32.262 100 1113	6.287 100 53	3.253 100 22	4.055 100 28	14.469 100 94

MP: mortalidade proporcional em %; TxM: taxa de mortalidade por 100 mil habitantes; DAC: doenças do aparelho circulatório; MC: malformações congênitas; PCR: parada cardiorrespiratória.

**Figura 1:**
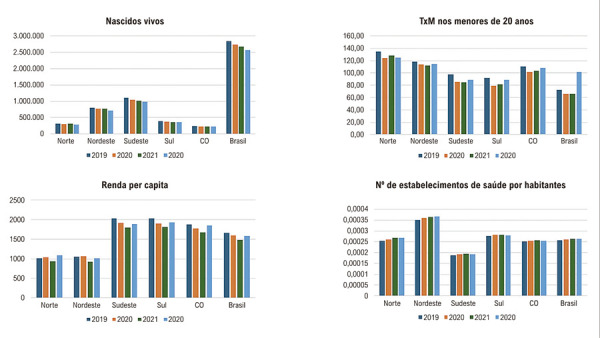
– Tendência no número de nascidos vivos, taxa de mortalidade nos menores de 20 anos, renda per capita e estabelecimentos de saúde por habitantes, no Brasil e regiões de 2019 a 2022.

Na Figura 1, observamos comparativamente o número de nascidos vivos, taxa de mortalidade em menores de 20 anos, renda per capita e número de estabelecimentos de saúde por habitantes no Brasil e regiões no mesmo período estudado. O número de nascidos vivos de 2019 a 2022 diminuiu em todas as regiões. As maiores taxas de mortalidade são encontradas nas regiões Norte e Nordeste, onde também existem as menores rendas, com aumento das taxas de mortalidade nos anos em que a renda diminui. Nas regiões com maiores rendas per capita, Sul e Sudeste, se encontram as menores taxas de mortalidade. A região com maior número de estabelecimentos de saúde, incluindo centros de atendimento de urgência, hospitais gerais, hospitais especializados, postos de saúde e unidades de atenção primária, é a Nordeste, sendo a região Sudeste a que apresenta menor número.

Os dados de renda per capita e RM foram analisados usando um modelo estatístico de regressão linear, conforme apresentado no Figura Suplementar, com 20 observações e contabilização por 68,87% da variação total. Obteve-se uma estatística F de 39,8 e um valor p de 0, ambos altamente significativos, confirmando uma relação negativa entre renda e RM nessas regiões.

Descrevem-se, na Tabela 2, as taxas de mortalidade por causas básicas de morte, na mesma faixa etária e período, por regiões federativas brasileiras. Reafirma-se que as principais causas básicas de morte nos menores de 20 anos entre todas as causas foram: as perinatais, externas e malformações congênitas, as que estão distribuídas entre os 4°, 3° e 2° quartis. A maior TxM ocorreu na região Norte em 2021, com 39 mortes por causas perinatais por 100 mil habitantes.

**Tabela 2:** – Taxa de mortalidade por causas básicas de morte nos menores de 20 anos, no Brasil e suas regiões federativas, em quartis, nos anos de 2019, 2020 e 2021

CAUSAS BÁSICAS	BRASIL				SUDESTE				SUL				CENTRO-OESTE				NORTE				NORDESTE			
	2019	2020	2021	2022	2019	2020	2021	2022	2019	2020	2021	2022	2019	2020	2021	2022	2019	2020	2021	2022	2019	2020	2021	2022
**Infecciosas e parasitárias**	2,8	3,4	3,9	5,7	3,5	3,9	4,5	4,6	2,4	2,5	2,5	4,1	4,4	4,6	5,6	6,4	6,7	9,9	9,6	8,8	4,7	6,3	6,9	6,7
**Neoplasias e sangue**	3,7	3,2	3,4	5,1	5,1	4,4	4,7	4,7	5,3	4,1	4,6	4,9	5,3	5,1	4,9	6,1	6,2	5,8	6,1	5,8	5,8	5,1	5,5	5,3
**Endócrinas e sistema nervoso**	4,0	3,2	3,4	6,3	5,7	4,3	4,7	5,9	5,0	4,1	4,6	5,7	7,0	6,0	5,4	6,7	7,1	5,7	6,4	7,4	6,1	5,6	5,2	6,6
**Sistema respiratório**	4,1	2,4	2,6	6,7	5,5	3,3	3,7	5,6	3,5	1,9	2,2	4,2	6,2	3,3	3,9	7,0	10,8	6,3	7,2	10,8	6,2	3,8	3,6	7,5
**Perinatal**	22,6	21,0	20,8	30,3	31,7	28,2	27,8	28,1	28,7	25,7	25,3	26,2	32,5	30,1	30,8	29,9	38,0	37,7	39,0	34,4	36,8	35,0	35,1	33,5
**DAC**	1,6	1,4	1,4	2,4	2,4	2,3	2,1	2,4	1,7	1,3	1,4	1,6	1,7	2,2	2,3	2,7	2,7	2,3	2,3	2,4	2,9	2,1	2,3	2,5
**MC**	11,0	9,3	9,4	14,9	15,1	13,0	12,9	13,8	16,4	14,2	14,4	15,5	18,4	16,1	16,1	17,4	17,6	14,5	15,8	16,0	16,4	13,9	14,2	14,8
**Externas**	18,1	18,0	16,6	23,8	21,6	19,9	18,1	17,7	23,8	21,4	22,1	21,7	28,7	29,1	27,3	25,0	34,4	37,7	31,3	28,9	32,2	35,3	31,9	30,6
**Outras causas**	2,0	1,7	1,9	3,0	2,6	2,2	2,4	2,7	2,1	1,7	1,7	1,9	3,5	2,2	3,0	3,4	4,5	4,7	4,5	4,2	3,4	2,9	3,2	3,4
**Mal definidas**	2,7	2,5	2,5	3,5	4,2	3,8	3,5	3,4	2,7	1,9	2,5	2,6	2,4	3,3	3,8	3,2	6,2	5,6	6,1	6,0	3,8	3,9	3,9	3,2

***Legenda:**
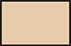
 1º Quartil – 25% 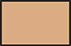 2º Quartil – 50% 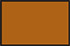 3º Quartil – 75% 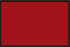 4º Quartil – 100%*

Na Tabela 2, entre as causas perinatais, é possível observar uma tendência de redução das TxM entre os anos de 2019 e 2021 no Brasil, com posterior aumento em 2022, o que também se observa nas regiões Sudeste e Sul. Já nas regiões Centro-Oeste, Norte e Nordeste, as TxM por causas perinatais seguem diminuindo em 2022. As causas externas de morte também apresentaram redução de TxM entre os anos 2019 e 2021 no Brasil, com posterior aumento em 2022, mas seguiu diminuindo nas suas regiões. Ocorreu redução na TxM por malformações congênitas, terceira maior causa de morte nessa população, entre os anos de 2019 e 2021, com posterior aumento em 2022 no Brasil e suas regiões. Neste mesmo período, evidenciou-se aumento nas TxM de doenças infecto-parasitárias em todas as regiões, entre 2019 e 2021, com posterior aumento em 2022, com exceção das regiões Norte e Nordeste que apresentaram redução no último ano na TxM por esta causa. As causas respiratórias apresentaram redução entre 2019 e 2021, com expressivo aumento em 2022 no Brasil e regiões.

**Figura 2:**
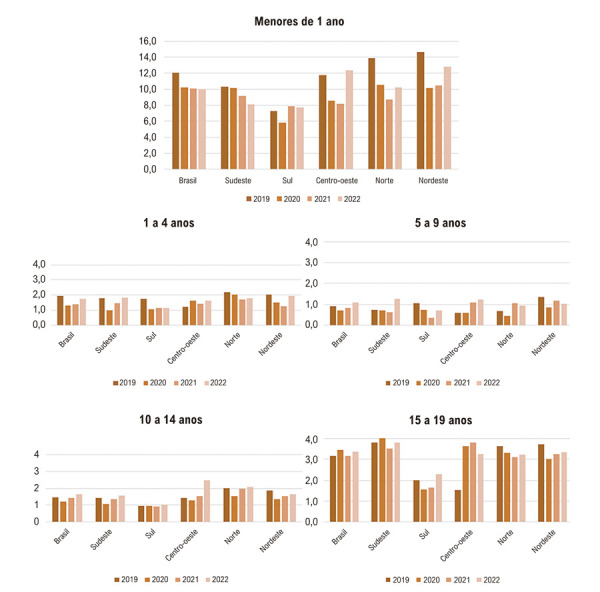
– Taxa de mortalidade por DAC no Brasil e suas regiões, em ambos os sexos, por faixa etária.

Na Figura 2, se apresenta a taxa de mortalidade por doenças do aparelho circulatório entre os anos de 2019 e 2022, no Brasil e suas regiões, por faixas etárias. Nos menores de 1 ano, observamos maiores taxas de óbito por esta causa nas regiões Nordeste, Norte e Centro-Oeste, com redução na mortalidade no Brasil e Sudeste no período e aumento nas outras regiões entre 2021 e 2022. Nas faixas etárias de 1 a 4, 5 a 9 e 10 a 14 anos encontramos diminuição nas mortes entre 2019 e 2021, com posterior aumento em 2022. Na faixa etária de 15 a 19 anos, se observa aumento na TxM de mortalidade por DAC nos anos de 2020 e 2021 no Brasil e nas regiões Sudeste e Centro-Oeste, com padrão de redução entre 2020 e 2021 e posterior aumento em 2022 nas demais regiões.

**Figura 3:**
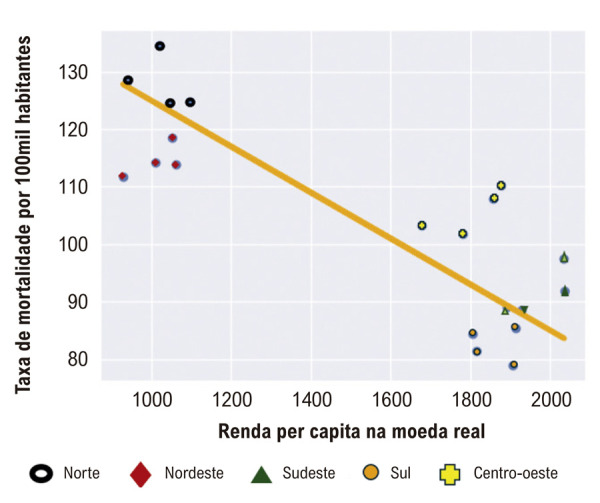

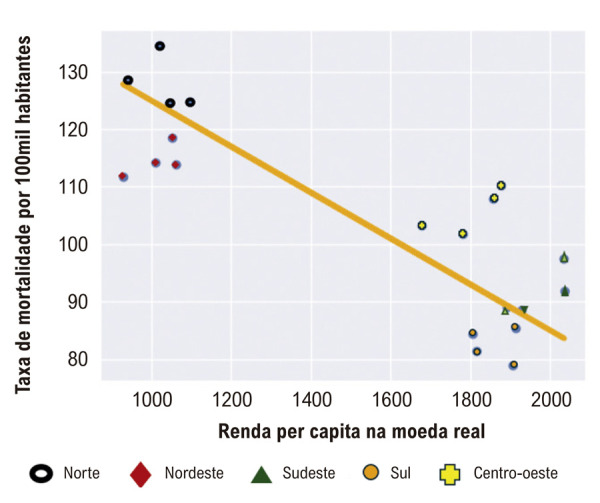
– Modelo estatístico de regressão PanelOLS com dados fixos, da correlação da taxa de mortalidade por todas as causas em menores de 20 anos por macrorregião Brasileira de 2019-2022.

A correlação dos dados de renda per capita e taxa de mortalidade por todas as causas apresentaram PanelOLS com dados fixos, descritos na Figura 3, com 68,87% de variação total explicada pelo modelo. Foi encontrado um F-estatístico de 39,8 e um p-valor de 0 (intervalo de confiança 95%: 0,0426 e -0,0213). E a correlação foi de que a cada aumento de 1 unidade na Renda, a Taxa de mortalidade diminui em 0.032 unidades, mantidas constantes as diferenças entre regiões (por causa do efeito fixo). Indicando uma correlação negativa entre morte e taxa de óbito por qualquer causa na infância. A região Norte apresentou menores valores de renda per capita com maiores taxas de mortalidade por todas as causas entre os menores de 20 anos e o inverso foi observado na região Sul que tem maiores valores de renda per capita com menores taxas de mortalidade entre os menores de 20 anos.

## Discussão

No Brasil, durante o período de 2019 a 2022, a taxa de mortalidade nos menores de 20 anos por todas as causas básicas baixou, voltando a uma tendência de crescimento em 2022, sendo as principais causas de morte as perinatais, externas e malformações congênitas, em ambos os sexos. Essa redução foi consequência do decréscimo na taxa de mortalidade das principais causas de morte dessa população nos anos de 2019 a 2021, com tendência de crescimento em 2022. No mesmo período, ocorreu a redução no número de nascidos vivos em todo o país. A taxa de mortalidade por doenças do aparelho circulatório aumentou na faixa etária de 15 a 19 anos entre 2020 e 2021, diminuindo nas outras faixas etárias. A taxa de mortalidade por todas as causas se apresentou mais expressiva em regiões de menor renda per capita, tendo uma aparente correlação, com seus valores flutuando em sincronia. O número de estabelecimentos de saúde por habitante por região não pareceu apresentar associação com as taxas de mortalidade.^12^

A taxa de mortalidade infantil no Brasil se reduziu cerca de 80% nos últimos 30 anos, saindo de 52 mortes por 1000 nascidos vivos em 1990 para 13,3 mortes em 2022.^4^ Isso se deveu a melhores condições de saúde no país nos últimos anos, principalmente após a implantação do Sistema Único de Saúde em 1980, e com os avanços socioeconômicos e na medicina. Um estudo ecológico de séries temporais realizado entre 1998 e 2008 mostrou que, nesse período, a cobertura pela estratégia saúde da família aumentou no Brasil, acompanhando a queda da mortalidade infantil em todo o país; as regiões Norte e Nordeste continuaram apresentando as maiores taxas de mortalidade, o que os autores atribuíram às históricas desigualdades sociais nessas áreas.^13^ Sendo assim, as disparidades de renda e IDH nas regiões macroeconômicas no Brasil ainda são motivo de preocupação e seus reflexos na saúde pública podem ser evidenciados nos estudos sobre a mortalidade infantil.

Também existe uma preocupação com o impacto da pandemia de COVID-19 na taxa de mortalidade nas crianças e adolescentes, e a influência das disparidades socioeconômicas das regiões federativas no número de mortes. Embora no mundo as crianças e adolescentes terem sido menos afetados por essa doença,^14^ os estudos aprofundando o impacto nas causas básica de morte dessa população nesse período são limitados no Brasil.

Nesse contexto, a doença do aparelho circulatório, mais especificamente a doença cardiovascular, se mostrou como um dos principais fatores de risco para um pior prognóstico na COVID-19 e para evolução a óbito em adultos, como evidenciado em estudos.^10^ No Brasil, nos menores de 15 anos a taxa de mortalidade por DAC se reduziu durante todo o período da pandemia, apresentando uma tendência de aumento no ano de 2022. Esse fato pode estar associado a menor diagnóstico e tratamento precoce das DAC durante o período e por possível redução do acesso à assistência médica no contexto de emergência em saúde. Entretanto, nos maiores de 15 anos encontramos um aumento na taxa de mortalidade por DAC no Brasil, principalmente nas regiões Sudeste e Centro-Oeste, o que pode estar relacionado ao impacto da DAC como fator de risco para óbito por COVID-19 nesta população, apesar de não encontrarmos estudos que corroborem essa associação nessa faixa etária.

As disparidades socioeconômicas e o impacto na taxa de mortalidade durante a pandemia foi motivo de estudo no Rio de Janeiro.^15^ A análise da coorte utilizada evidenciou que moradores de favelas, locais de maior vulnerabilidade social, apresentaram maior taxa de mortalidade por COVID-19, seja por menor capacidade de isolamento social por precarização de moradias ou por menor acesso a assistência médica. Outro estudo ecológico, realizado no Brasil,^16^ com população na faixa etária de 0 a 14 anos, evidenciou maiores taxas de mortalidade nos residentes das regiões Norte e Nordeste, assim como no presente estudo, o que provavelmente está relacionado às desigualdades socioeconômicas.

A análise dos dados selecionados para este estudo mostrou que as regiões Norte e Nordeste apresentaram o maior número de estabelecimentos de saúde per capita, apesar de apresentarem maiores taxas de mortalidade por causas básicas (não foi analisada a distribuição desses estabelecimentos entre os municípios e suas microrregiões). Esse fato pode estar relacionado à qualidade desse serviço, distribuição dos recursos e acesso geográfico e logístico, o que demanda maiores análises e melhoria da capacitação de profissionais e políticas públicas voltadas para áreas remotas e populações mais vulneráveis.

O declínio no número de nascidos vivos no Brasil refletiu uma tendência global. Houve declínios em países como Itália, Espanha e Hungria, com muitos deles registrando menos nascimentos do que mortes no mesmo ano, semelhante ao observado nos Estados Unidos. Esse padrão pode revelar como emergências de saúde impactam os hábitos de vida e o planejamento da fertilidade da população, fenômeno observado durante a pandemia de gripe espanhola, conforme ilustrado em outro estudo brasileiro.^17,18^Dados de qualidade e análises de conclusão de DC resultaram em grandes esforços de melhoria no Brasil na última década, com foco em uma conclusão mais precisa pelas partes que atestam e na redução das menções de causas mal definidas e códigos de lixo como causa básica de morte.^11,12^ Esperava-se que, com o advento da pandemia de COVID-19 e todas as problemáticas correlatas a um momento de caos em saúde pública, o número de causas mal definidas, e nesse caso a parada cardiorrespiratória, pudesse se tornar mais frequente como causa básica de óbito atestada nas crianças e adolescentes. Entretanto, não foi o que constatamos neste estudo, reforçando que o tempo dispendido em pesquisa e preparo dos profissionais atestantes foi notoriamente necessário, aumentando a qualidade dos dados de declarações de óbito no país, mesmo em um contexto de emergência pública.

Este estudo apresenta limitações devido às variações na qualidade do preenchimento da DO, o que, por vezes, resulta em informações omitidas, incompletas ou classificadas incorretamente. Isso pode causar problemas na análise dos dados e a perda de detalhes sobre o local dos óbitos, apesar das melhorias significativas nos últimos anos, conforme demonstrado nos resultados e já discutido. Mesmo assim, o SIM é a melhor fonte de dados de mortalidade no Brasil, um recurso crucial para o estudo e a discussão da vigilância em saúde e epidemiológica no país.

## Conclusão

A hipótese de que haveria um aumento no número de mortes nos menores de 20 anos no Brasil durante a pandemia não se sustentou, notando-se um padrão semelhante aos países de alta renda que apresentaram redução na taxa de mortalidade nesta população entre 2019 e 2022, assim como na taxa de nascidos vivos. As maiores taxas de mortalidade por causas básicas foram encontradas nas regiões Norte e Nordeste, regiões essas de menor renda per capita, apesar do Nordeste apresentar o maior número de estabelecimentos de saúde por habitante. Nos maiores de 15 anos, a taxa de mortalidade por doenças do aparelho circulatório aumentou durante a pandemia, enquanto a descrição da PCR nas DO se reduziu em todas as faixas etárias.
