# Supplementary material for: Perfil de Morte por Todas as Causas e o Comportamento das Doenças do Aparelho Circulatório na Infância entre 2019 e 2022 no Brasil
Source: Arq Bras Cardiol. 2025 Dec 22;122(12):e20250191. [Article in Portuguese] doi: 10.36660/abc.20250191 (PMC12978379; doi:10.36660/abc.20250191)
Supplement: Translation

# Translation: Profile of Mortality Due to All Causes and Patterns of Diseases of the Circulatory System in Childhood Between 2019 and 2022 in Brazil

**DOI:** 10.36660/abc.20250191i

**Authors:** Thayanne Mendes de Andrade, Mariara Lopes da Costa Marques, Julia Pereira Cavalcante Marques, Maria Eduarda Miranda de Souza, Gláucia Maria Moraes de Oliveira, Thais Rocha Salim

**Affiliations:** 1 Brazil Universidade Federal do Rio de Janeiro, Rio de Janeiro, RJ – Brazil; 2 Brazil Universidade de Vassouras, Vassouras, RJ – Brazil

**Keywords:** Cardiovascular Diseases, Child Mortality, Heart Arrest

## Abstract

**Background:**

Between 2020 and 2023, the COVID-19 pandemic had a direct impact on population health. In Brazil, there is a lack of studies on the correlation between mortality from diseases of the circulatory system (DCS) and cardiorespiratory arrest (CRA) among people younger than 20 years during this period.

**Objective:**

To analyze, using DCS and CRA, the mortality rates (MRs) due to underlying causes of death among people younger than 20 years in Brazil from 2019 to 2022 to understand the impact of the pandemic.

**Methods:**

Ecological time-series study conducted between 2019 and 2022, focusing on deaths among people younger than 20 years. The analysis included the MRs and proportional mortality (PM) by cause of death, with data obtained from the DATASUS. Stata and Excel were used for statistical analysis.

**Results:**

Between 2019 and 2022, there were 243,358 deaths among people younger than 20 years, with an MR of 101.7 per 100,000 inhabitants, which was higher among children younger than 1 year. The main causes of death were perinatal, external, and congenital malformations. There was a 9% reduction in MR between 2019 and 2020, followed by a 4.08% increase between 2021 and 2022, along with a 10% decrease in the number of live births. The highest MRs were in the North and Northeast regions. The MRs by DCS decreased between 2019 and 2021 but increased in 2022. Reporting of CRA as the cause of death was infrequent.

**Conclusion:**

There was a reduction in MRs due to all causes and an increase in DCS in people older than 15 years during the pandemic, with disparities in lower-income regions.

## Introduction

Over the last two decades, mortality rates (MRs) have declined worldwide among people younger than 20 years. Brazil exhibited a similar trend, with a reduction in MR of more than 50%. This trajectory can be attributed to improved access to healthcare and education, as well as lower poverty and fertility rates.^1^ However, external causes of death, i.e*.*, those not caused by diseases but by environmental and perinatal factors, are still the main underlying causes of death in Brazil. These causes could be prevented or lessened through improvements in care for pregnant women and newborns, along with public health and educational measures to decrease social inequality and lower these deaths.

Between 2020 and 2023, the COVID-19 pandemic directly affected the population’s health, changing not only how we live but also how we become sick and die. An ecological, multicenter study involving eighteen low- and middle-income countries showed a 3.6% increase in infant mortality and a 1.5% rise in maternal mortality from 2020 to 2021, which the authors attributed to reduced access to healthcare and early diagnosis for eventual treatment.^2^ In contrast, a cohort study conducted during the same period in England, a high-income country, showed a decrease in infant mortality, which correlated with the decline in death rates from infectious diseases, likely due to social distancing and COVID-19 prevention measures that also reduced respiratory infectious diseases.^3^ Therefore, it is possible to observe a variation in infant MR patterns based on the socioeconomic profile across countries and, possibly, their strategies for dealing with the pandemic.

In Brazil, we found no studies correlating the infant MR and the trajectory of underlying causes of death in people younger than 20 years during the COVID-19 pandemic and the socioeconomic levels of their municipalities. Our hypothesis is that isolation and social restrictions, although necessary to contain the spread of the coronavirus, may have led to reduced access to healthcare in the country, with a harmful effect on the diagnosis and treatment of diseases, especially those that require more resources and personnel for diagnosis, such as diseases of the circulatory system (DCS), resulting in an increase in deaths in this age group during the period.

Furthermore, cardiovascular disease was identified as one of the major risk factors for a worse prognosis and progression to death in COVID-19.^4-10^ In Brazil, among adults, it was observed DCS as the underlying cause of death, and the description of cardiorespiratory arrest (CRA) as the cause of death decreased during the pandemic period, which is possibly related to reduced access to diagnosis and treatment, as well as potentially increased reporting of COVID-19 as the underlying cause of death.^11^ We found no studies examining the pattern of deaths caused by DCS in children and adolescents during the pandemic in the country.

Therefore, this study aimed to carry out a population analysis of MR by underlying causes of death in Brazil among people younger than 20 years in the years 2019, 2020, 2021, and 2022, and in both sexes, before and during the COVID-19 pandemic, along with the MR pattern in the country’s federative regions. Additionally, the study aimed to evaluate the profile of deaths due to DCS in the same period and age group, and the description of CRA as the underlying cause of death in death certificates (DC), to understand the impact of the pandemic on childhood mortality patterns.

## Methods

### Study design

Ecological time-series study of deaths in people younger than 20 years, assessing MRs and proportional mortality (PM) by underlying cause of death in both sexes in Brazil from 2019 to 2022. The analysis included the distribution of death rates by quartiles based on underlying causes, the MR from DCS, and DC mentions of CRA in this age group and period across the country’s federative regions. It also examined trends of live births, per capita income, the number of healthcare facilities per inhabitant, and the all-cause MR. The study was conducted in accordance with the principles of ethics. Since the study drew data from anonymized national databases available on the DATASUS website, it was exempt from approval by the research ethics committee in accordance with resolution 466/2012.

### Data source

Data on deaths were sourced from the Department of Informatics of the Unified Health System (DATASUS) website via the Mortality Information System (SIM).^4^ The data comprised sets of all DC registered in Brazil from 2019 to 2022, year by year, for each Brazilian state. The underlying cause of death was coded according to the 10th Revision of the International Statistical Classification of Diseases and Related Health Problems (ICD-10) from the World Health Organization.^8^ All files were converted for analysis using the software Tab for Windows, version 4.15, from DATASUS. The data were collected for both sexes and in the age groups of (1) under 1 year, (2) 1 to 4 years, (3) 5 to 9 years, (4) 10 to 14 years, and (5) 15 to 19 years, following the standards proposed by the World Health Organization.^5^

The population data used to calculate the MRs were projections derived from statistical calculations conducted by the Brazilian Institute of Geography and Statistics (IBGE),^6^ based on census data, available from 1980 to 2050 by Brazilian macroregion, sex, age group, and overall population. The study used projections from 2019 to 2022 for age groups 0 to 4 years (excluding live births during the period), 5 to 9 years, 10 to 14 years, and 15 to 19 years, covering both sexes and all Brazilian states. For children younger than 1 year, the number of live births available in the Live Birth Information System (SINASC)^7^ was used.

Information on per capita income was obtained from the IBGE website, based on the Continuous National Household Sample Survey conducted in 2022, with data available year by year from 2019 to 2022 and by federative region.

Data on healthcare facilities were obtained from the DATASUS website through the National Registry of Health Establishments (CNES), displayed on TABNET, year by year, between 2019 and 2022, by federative region. These healthcare facilities provide services to all age groups without distinction.

### Data analysis

The MRs per 100,000 inhabitants and PM by underlying cause of death in people younger than 20 years, by sex and age group, for the years 2019, 2020, 2021, and 2022, were calculated. The MRs by underlying cause of death were also calculated for people younger than 20 years, by sex and age group, and distributed across the North, Northeast, South, Southeast, and Central-West regions, and their quartiles, presented in tables. The mentions of CRA as the underlying cause of death were calculated, along with its MRs and PM, and presented in a table for analysis of its occurrence, despite being considered garbage code.

Quartiles were calculated for each year based on the MR percentiles for people younger than 20 years, by underlying causes of death, in Brazil and each federative region. They were divided among the 25%, 50%, 75%, and 100% lowest MRs during the period and analyzed year by year.

The MRs due to DCS in Brazil and its regions were analyzed separately for both sexes, by age groups, along with the trend of live births during the same period, and the MRs from all underlying causes of death in people younger than 20 years.

A statistical analysis model was built in PanelOLS with fixed data, used for time-series regression. The dependent variable was the all-cause mortality rate for individuals under 20 years of age by Brazilian macroregion, year. The independent variable was per capita income by macroregion and year. With this information, we can infer that the objective of the analysis is to investigate the effect of per capita income on the mortality rate for individuals under 20 years of age in different regions of Brazil, taking into account the fixed characteristics of each region and the temporal evolution of the data. The independent variable was per capita income.

The software used was Microsoft Excel^8^ and Stata, version 14.^9^

## Results

As shown in the Central Illustration, there were 243,358 deaths due to all underlying causes among people younger than 20 years across both sexes in Brazil from 2019 to 2022, with an MR of 101.7 per 100,000 inhabitants during the period. This rate was higher among children younger than 1 year, regardless of the underlying cause of death. The main causes of death were perinatal, external, and congenital malformations, respectively. The MR decreased by 9% between 2019 and 2020 and by 0.1% between 2020 and 2021, then increased by 4.08% between 2021 and 2022. The number of live births throughout the period declined by 10%. The highest MRs occurred in the North and Northeast regions. The MR from DCS generally declined in Brazil and its regions between 2019 and 2021, with some exceptions, before rising again in 2022. Mentions of CRA in DC throughout the period were not prominent in any age group, with MRs ranging between 0.01 and 0.03 per 100,000 inhabitants during the period.

Table 1 shows the MRs and PM in Brazil categorized by age group and underlying cause of death. During the four-year observation period, the main causes of death were perinatal, external, and congenital malformations, with the highest MR being 1,238.9 per 100,000 inhabitants in 2019 for all causes in children younger than 1 year, which was followed by a progressive decrease over the subsequent years, with congenital malformations being the second condition with the highest MR in this age group. The second age group with the highest MR was 15 to 19 years, with the most notable MR being 105.3 per 100,000 inhabitants in 2020 for all underlying causes of death, which was higher than in 2019, with external causes being the most prominent. Across all other age groups, external causes were the leading cause of death, except for the year 2022, when respiratory diseases were the main cause of death in the age group of 1 to 4 years.

**Table 1:** – Underlying causes of death, by age group, among those under 20 years of age, from 2019 to 2022, for both sexes, in Brazil

Underlying causes of death		2019						2020						2021						2022					
		TOTAL	< 1	1-4	5-9	10-14	15-19	TOTAL	<1	1-4	5-9	10-14	15-19	TOTAL	<1	1-4	5-9	10-14	15-19	TOTAL	<1	1-4	5-9	10-14	15-19
**Infectious**	Deaths PM MRs	2,512 4 3	1,263 4 44	467 8 4	202 6 1	198 5 1	382 2 2	3,086 5 3	1,381 4 50	537 12 4	247 9 1	271 7 2	650 4 4	3,484 6 4	1,432 4 53	570 11 5	254 9 2	341 9 2	887 6 6	3,406 5 6	1,608 5 55	688 11 6	288 9 2	305 7 2	517 3 3
**Neoplasms and Blood**	Deaths PM MRs	3,312 5 4	326 1 11	663 11 6	683 22 4	670 16 4	970 6 6	2,889 5 3	251 1 9	553 12 4	570 22 4	645 16 4	870 5 5	3,029 5 3	286 1 11	625 12 5	612 22 4	610 16 4	896 6 6	3,041 5 5	291 1 10	654 10 5	613 19 4	616 15 4	867 6 5
**Endocrine and Nervous System**	Deaths PM MRs	3,560 5 4	753 2 26	783 13 7	550 17 4	610 14 4	864 5 5	2,911 5 3	646 2 23	542 12 4	347 13 2	555 14 4	821 5 5	3,041 5 3	639 2 24	653 13 5	411 15 3	514 13 3	848 5 5	3,755 6 6	693 2 24	857 13 7	563 17 4	639 16 4	1,003 7 6
**Respiratory System**	Deaths PM MRs	3,694 6 4	1,627 4 57	1,013 17 9	277 9 2	300 7 2	477 3 3	2,165 3 2	829 2 30	479 10 4	164 6 1	203 5 1	490 3 3	2,302 4 3	1,012 3 38	524 10 4	179 6 1	188 5 1	399 2 2	3,951 6 6	1,600 5 55	1,232 19 10	317 10 2	298 7 2	504 3 3
**Perinatal**	Deaths PM MRs	20,291 31 23	20,213 57 709	42 1 0	14 0 0	7 0 0	15 0 0	18,665 31 21	18,618 59 682	24 0 0	8 0 0	3 0 0	12 0 0	18,510 31 21	18,468 58 691	24 0 0	8 0 0	2 0 0	8 0 0	17,935 30 30	17,869 55 617	31 0 0	11 0 0	9 0 0	15 0 0
**DCS**	Deaths PM MRs	1,476 2 2	344 1 12	219 4 2	137 4 1	236 6 1	540 3 3	1,267 2 1	279 1 10	154 3 1	104 4 1	181 4 1	549 3 3	1,261 2 1	270 1 10	164 3 1	121 4 1	211 5 1	495 3 3	1,413 2 2	289 1 10	207 3 2	160 5 1	241 6 1	516 3 3
**CM**	Deaths PM MRs	9,799 15 11	8,396 24 295	812 14 7	212 6 1	186 4 1	193 1 1	8,318 14 9	7,273 23 266	594 13 5	149 6 1	153 4 1	149 1 1	8,374 14 9	7,311 23 273	621 12 5	177 6 1	117 3 1	148 1 1	8,816 14 15	7,399 23 255	772 12 6	254 8 2	180 4 1	211 1 1
**External**	Deaths PM MRs	16,310 25 18	981 3 34	1,209 21 11	736 23 5	1,549 36 9	11,835 71 70	16,023 27 18	863 3 31	1213 26 10	752 29 5	1,445 37 10	11,750 70 74	14,797 25 16	1,034 3 39	1,249 25 10	708 25 5	1,353 36 9	10,453 67 67	14,107 23 24	1,183 3 41	1,208 19 10	711 22 5	1,313 32 9	9,692 67 63
**Other Causes**	Deaths PM MRs	1,847 3 2	500 1 17	258 4 2	184 6 1	244 6 4	661 4 4	1,559 2 2	469 1 17	215 4 2	122 5	208 5 1	545 3 3	1,675 3 2	404 1 15	253 5 2	135 5 1	233 6 1	650 4 4	1,804 3 3	486 1 17	294 4 2	176 5 1	261 6 2	587 4 4
**Ill-Defined**	Deaths PM MRs	2,405 4 3	890 2 31	356 6 3	171 5 1	224 5 1	764 4 4	2,246 4 2	830 2 30	275 6 2	119 4 1	216 5 1	806 5 5	2,262 4 2	874 3 33	302 6 2	163 6 1	208 5 1	715 4 4	2,070 3 3	839 2 29	340 5 3	157 5 1	190 4 1	544 4 3
**CRA**	Deaths PM MRs	7 0 0	0 0 0	2 0 0	0 0 0	1 0 0	4 0 0	20 0 0	7 0 0	1 0 0	0 0 0	3 0 0	9 0 0	20 0 0	7 0 0	0 0 0	2 0 0	3 0 0	8 0 0	28 0 0	5 0 0	4 0 0	3 0 0	3 0 0	13 0 0
**TOTAL**	Deaths PM MRs	65,213 100 107	35,293 100 1226	5,824 100 47	3166 100 20	4225 100 27	16,705 100 100	59,149 100 98	31,446 100 1117	4,587 100 37	2,582 100 17	6,465 100 24	16,651 100 98	58,755 100 98	31,737 100 1076	4,985 100 42	2,770 100 19	3,780 100 25	15,507 100 99	60,326 100 102	32,262 100 1113	6,287 100 53	3,253 100 22	4,055 100 28	14,469 100 94

DCS: diseases of the circulatory system; CM: congenital malformations; CRA: cardiorespiratory arrest; PM: proportional mortality; MRs: mortality rates.

Figure 1 shows a comparison of the number of live births, MRs among people younger than 20 years, per capita income, and the number of healthcare facilities per person in Brazil and its regions during the study period. The number of live births decreased in all regions from 2019 to 2022. The highest MRs were observed in the North and Northeast regions, which also had the lowest incomes, with MRs increasing during years when income decreased. The lowest MRs were observed in the South and Southeast regions, which have the highest per capita income. The region with the largest number of healthcare facilities, including urgent care centers, general hospitals, specialized hospitals, health stations, and primary care units, is the Northeast, with the Southeast region having the lowest number.

**Figure 1:** – Trend in the number of live births, mortality rate in those under 20 years of age, per capita income, and health establishments per inhabitant, in Brazil and regions from 2019 to 2022.

The per capita income and MR data were analyzed using a linear regression statistical model, as presented in the Supplementary Figure, with 20 observations and accounting for 68.87% of the total variation. An F-statistic of 39.8 and a p-value of 0 were obtained, both highly significant, confirming a negative relationship between income and MR in these regions.

Table 2 presents the MRs by underlying causes of death by age group and period, categorized by Brazilian federative region. It confirms that the main underlying causes of death in people younger than 20 years across all causes were perinatal, external, and congenital malformations, which are distributed between the 4th, 3rd, and 2nd quartiles. The highest MR was in the North region in 2021, with 39 perinatal deaths per 100,000 inhabitants.

**Table 2:** – Mortality rate by underlying causes of death in individuals under 20 years of age in Brazil and its federal regions, in quartiles, in 2019, 2020, and 2021

UNDERSTANDING CAUSES	BRAZIL				SOUTHEAST				SOUTH				CENTRAL-WEST				NORTH				NORTHEAST			
	2019	2020	2021	2022	2019	2020	2021	2022	2019	2020	2021	2022	2019	2020	2021	2022	2019	2020	2021	2022	2019	2020	2021	2022
**Infectious and Parasitic**	2.8	3.4	3.9	5.7	3.5	3.9	4.5	4.6	2.4	2.5	2.5	4.1	4.4	4.6	5.6	6.4	6.7	9.9	9.6	8.8	4.7	6.3	6.9	6.7
**Neoplasms and Blood**	3.7	3.2	3.4	5.1	5.1	4.4	4.7	4.7	5.3	4.1	4.6	4.9	5.3	5.1	4.9	6.1	6.2	5.8	6.1	5.8	5.8	5.1	5.5	5.3
**Endocrine and Nervous System**	4.0	3.2	3.4	6.3	5.7	4.3	4.7	5.9	5.0	4.1	4.6	5.7	7.0	6.0	5.4	6.7	7.1	5.7	6.4	7.4	6.1	5.6	5.2	6.6
**Respiratory System**	4.1	2.4	2.6	6.7	5.5	3.3	3.7	5.6	3.5	1.9	2.2	4.2	6.2	3.3	3.9	7.0	10.8	6.3	7.2	10.8	6.2	3.8	3.6	7.5
**Perinatal**	22.6	21.0	20.8	30.3	31.7	28.2	27.8	28.1	28.7	25.7	25.3	26.2	32.5	30.1	30.8	29.9	38.0	37.7	39.0	34.4	36.8	35.0	35.1	33.5
**DCS**	1.6	1.4	1.4	2.4	2.4	2.3	2.1	2.4	1.7	1.3	1.4	1.6	1.7	2.2	2.3	2.7	2.7	2.3	2.3	2.4	2.9	2.1	2.3	2.5
**CM**	11.0	9.3	9.4	14.9	15.1	13.0	12.9	13.8	16.4	14.2	14.4	15.5	18.4	16.1	16.1	17.4	17.6	14.5	15.8	16.0	16.4	13.9	14.2	14.8
**External**	18.1	18.0	16.6	23.8	21.6	19.9	18.1	17.7	23.8	21.4	22.1	21.7	28.7	29.1	27.3	25.0	34.4	37.7	31.3	28.9	32.2	35.3	31.9	30.6
**Other Causes**	2.0	1.7	1.9	3.0	2.6	2.2	2.4	2.7	2.1	1.7	1.7	1.9	3.5	2.2	3.0	3.4	4.5	4.7	4.5	4.2	3.4	2.9	3.2	3.4
**Ill-Defined**	2.7	2.5	2.5	3.5	4.2	3.8	3.5	3.4	2.7	1.9	2.5	2.6	2.4	3.3	3.8	3.2	6.2	5.6	6.1	6.0	3.8	3.9	3.9	3.2

Legend:  1^st^ Quartile – 25%  2^nd^ Quartile – 50%  3^rd^ Quartile – 75%  4^th^ Quartile – 100%

Among the perinatal causes, there was a noticeable decrease in MRs from 2019 to 2021 in Brazil, followed by an increase in 2022, which is also seen in the Southeast and South regions, as shown in Table 2. In the Central-West, North, and Northeast regions, the MRs attributed to perinatal causes continued to decline in 2022. The MRs due to external causes of death also decreased over the years in Brazil, followed by an increase in 2022, but it continued to decline across the regions. Between 2019 and 2021, there was a reduction in MRs from congenital malformations, the third leading cause of death in this population, followed by a subsequent increase in 2022 in Brazil and its regions. During the same period, there was an increase in MRs due to infectious and parasitic diseases in all regions, from 2019 to 2021, followed by a further increase in 2022, except for the North and Northeast regions, which experienced a reduction in MR for this cause in the last year. Respiratory causes decreased between 2019 and 2021 and increased prominently in 2022 in Brazil and its regions.

Figure 2 illustrates the MR from DCS in Brazil and its regions between 2019 and 2022, categorized by age group. In people younger than 1 year, we observed higher death rates from this cause in the Northeast, North, and Central-West regions, with a reduction in mortality across the country and in the Southeast during this period, and an increase in other regions between 2021 and 2022. In the age groups of 1 to 4, 5 to 9, and 10 to 14 years, we observed a decrease in deaths between 2019 and 2021, followed by a subsequent increase in 2022. In the age group of 15 to 19 years, an increase in MRs due to DCS was observed in 2020 and 2021 across the country and specifically in the Southeast and Central-West regions, with a trend of reduction between 2020 and 2021 and a subsequent increase in 2022 in the other regions.

**Figure 2:** – Mortality rate due to coronary artery disease in Brazil and its regions, for both sexes, by age group.

The correlation of per capita income data and mortality rate from all causes presented PanelOLS with fixed data, described in Figure 3, with 68.87% of the total variation explained by the model. An F-statistic of 39.8 and a p-value of 0 (95% confidence interval: -0.0426 and -0.0213) were found. The correlation was that for every 1-unit increase in income, the mortality rate decreases by 0.032 units, keeping the differences between regions constant (because of the fixed effect). Indicating a negative correlation between death and mortality rate from any cause in childhood. The North region presented lower per capita income values with higher mortality rates from all causes among those under 20 years of age, and the opposite was observed in the South region, which has higher per capita income values with lower mortality rates among those under 20 years of age.

**Figure 3:** – PanelOLS regression statistical model with fixed data, of the correlation of the all-cause mortality rate among those under 20 by Brazilian macro-region, from 2019 to 2022.

## Discussion

In Brazil, during the period from 2019 to 2022, the MRs due to all underlying causes decreased in people younger than 20 years, then increased again in 2022, with the main causes of death being perinatal, external, and congenital malformations in both sexes. This decline was due to a reduction in MRs from the main causes of death in this population from 2019 to 2021, with a trend toward an increase in 2022. During the same period, there was a decline in the number of live births across the country. The MRs from DCS increased in the age group of 15 to 19 years between 2020 and 2021, while decreasing in other age groups. The all-cause MR was more pronounced in regions with lower per capita income, exhibiting an apparent correlation, with their values fluctuating in synchrony. The number of healthcare facilities per inhabitant per region did not appear to be associated with MRs.^12^

The infant MR in Brazil has dropped by about 80% over the past 30 years, decreasing from 52 deaths per 1,000 live births in 1990 to 13.3 deaths per 1,000 live births in 2022.^4^ This was due to improved health conditions in the country over the past years, especially after the implementation of the Unified Health System in 1980, along with socioeconomic and medical advancements. An ecological time-series study from 1998 to 2008 showed that, during this period, coverage by the family health strategy increased in Brazil, along with a decrease in infant mortality nationwide; the North and Northeast regions continued to have the highest death rates, which the authors attributed to the historical social inequalities in these areas.^13^ Therefore, disparities in income and the Human Development Index (HDI) in Brazil’s macroeconomic regions remain a concern, and their impact on public health is evident in studies on infant mortality.

There is also concern about the impact of the COVID-19 pandemic on the MRs of children and adolescents, and the influence of socioeconomic disparities across federative regions on the number of deaths. Although children and adolescents have been less affected by this disease worldwide,^14^ studies examining its impact on the underlying causes of death among this population during that period are limited in Brazil.

In this context, DCS, specifically cardiovascular disease, emerged as one of the main risk factors for a worse prognosis in COVID-19 and for progression to death in adults, as evidenced in studies.^10^ In Brazil, among people younger than 15 years, the MRs from DCS decreased during the pandemic period, with an upward trend observed in 2022. This fact may be linked to lower DCS diagnoses and earlier treatment during this time, possibly due to reduced access to medical care in a health emergency context. However, we found an increase in MRs due to DCS in Brazil in people older than 15 years, mainly in the Southeast and Central-West regions, which could be related to the impact of DCS as a risk factor for death from COVID-19 in this population. We found no studies corroborating such an association in this age group.

Socioeconomic disparities and their impact on MRs during the pandemic were examined in a study conducted in Rio de Janeiro.^15^ An analysis of the study cohort revealed that residents of favelas, areas with higher social vulnerability, experienced a higher MR from COVID-19, either because of limited ability to social distance due to inadequate housing or because of reduced access to healthcare. Another ecological study conducted in Brazil^16^involving a population aged 0 to 14 years showed higher MRs among residents of the North and Northeast regions, similar to the present study, which is probably related to socioeconomic inequalities.

The analysis of the data selected for this study showed that the North and Northeast regions had the highest number of healthcare facilities per capita, despite their higher MRs from underlying causes (the distribution of these facilities among the municipalities and their microregions was not analyzed). This fact may be related to the quality of the service, distribution of resources, and geographic and logistical access, which require further analysis and comparison to improve the training of professionals and public policies aimed at remote areas and more vulnerable populations.

The decline in the number of live births in Brazil mirrored a global trend. Declines were observed in countries such as Italy, Spain, and Hungary, with many experiencing fewer births than deaths in the same year, similar to the United States. This pattern can reveal how health emergencies impact a population’s lifestyle habits and fertility planning, a phenomenon observed during the Spanish flu pandemic, as illustrated in another Brazilian study.^17,18^

Quality data and DC completion analyses have resulted in major improvement efforts in Brazil over the last decade, focused on more accurate completion by attesting parties and reducing the mentions of ill-defined causes and garbage codes as the underlying cause of death.^11,12^ It was expected that, with the COVID-19 pandemic and all the related issues at a time of public health chaos, the number of ill-defined causes, and in this case CRA, would become more frequently mentioned as the underlying cause in DC of children and adolescents. However, this was not what the present study found, which highlights that the time spent on research and training of attesting professionals was definitely necessary and improved the quality of DC data in the country, even in the context of a public emergency.

This study has limitations due to variations in the quality of DC completion, which sometimes results in omitted, incomplete, or misclassified information. This can cause data analysis issues and the loss of details about the location of deaths, despite significant improvements in recent years, as shown in the results and already discussed. Even so, the SIM is the best source of mortality data in Brazil, a crucial resource for studying and discussing health and epidemiological surveillance in the country.

## Conclusion

The hypothesis that there would be an increase in the number of deaths among people under 20 years in Brazil during the pandemic was not supported. Instead, a pattern similar to that observed in high-income countries was seen, with a decrease in MRs in this age group between 2019 and 2022, along with a drop in live birth rates. The highest MRs from underlying causes were found in the North and Northeast regions, which have the lowest per capita income, despite the Northeast having the highest number of healthcare facilities per person. In those over 15 years old, mortality from DCS increased during the pandemic, while DC mentions of CRA decreased across all age groups.
